# Management of a Critically Ill Patient With Severe Hypertrophic Obstructive Cardiomyopathy Presenting for Emergent Craniotomy Due to Subdural Hemorrhage

**DOI:** 10.7759/cureus.20521

**Published:** 2021-12-19

**Authors:** Moez Mithani, Galila Flatow, Michael A Chyfetz

**Affiliations:** 1 Anesthesiology, Montefiore Medical Center, Bronx, USA

**Keywords:** obstruction, lvot, bleeding, management, hypertrophic, subdural, hemorrhage, cardiomyopathy, hcm, hocm

## Abstract

Hypertrophic obstructive cardiomyopathy (HOCM) is an inherited cardiac disorder characterized by asymmetric thickening of the myocardium, most commonly of the interventricular septum. Perioperative considerations for patients with HOCM undergoing surgical procedures are discussed, so as to avoid worsening the existing left ventricular outflow tract (LVOT) obstruction, leading to potential cardiovascular collapse. Of particular interest is managing these patients when confronted with vascular aneurysmal disease or hemorrhagic comorbidities with conflicting hemodynamic goals. In this case report, we present the case of a 77-year-old female with known HOCM and severe LVOT obstruction, presenting with intracranial hemorrhage (ICH), acute on chronic subdural hematoma, and potential cerebral aneurysm, undergoing decompressive craniectomy and hematoma evacuation. Anesthetic management of a patient with HOCM presenting for emergent ICH can be challenging given the complex hemodynamic management goals, often conflicting with a patient's comorbidities. Here we describe the prioritization of the patient's underlying HOCM pathology and favored maintenance of afterload given the potentially lethal risk of cardiovascular collapse if LVOT obstruction was encountered.

## Introduction

Hypertrophic cardiomyopathy (HCM) is an inherited cardiac disorder characterized by pathologic and asymmetrical thickening of the myocardium in the absence of an inciting factor or medical history [1]. The condition is predominantly seen as asymmetrical thickening of the interventricular septum. Histologically, the disease state results from disorganized cellular collagen deposition and aberrant interstitial fibrosis [2,3]. HCM is further subcategorized as obstructive or without obstruction. In hypertrophic obstructive cardiomyopathy (HOCM), the left ventricular outflow tract (LVOT) is obstructed, often by systolic anterior movement (SAM) of the anterior leaflet of the mitral valve [1,2,4]. Most often, only the anterior leaflet is involved in the LVOT obstruction. However, the posterior leaflet and chordal structures may also be involved. Because of the malcoaptation of the mitral leaflets, mitral regurgitation can also accompany HOCM physiology. 

While HCM can lead to functional impairment and overt clinical findings, the majority of patients remain asymptomatic [2,5]. Consequently, the diagnosis of HCM is typically made with echocardiography or cardiac magnetic resonance imaging [1,6-8]. The diagnostic criteria for HCM are met by the presence of left ventricular wall thickness ≥15 mm at any single point along the left ventricular wall. The diagnosis can also be made with a wall thickness of ≥13 mm in a patient with a positive family history of HCM [1]. 

After the diagnosis of HCM is made, risk stratification analysis is undertaken to determine the severity of the condition and the need for intervention and/or surveillance. This includes determining if the patient is symptomatic. While the majority of patients are asymptomatic, those who are symptomatic often initially present with signs of heart failure, chest pain, arrhythmias, syncope, and, rarely, with acute hemodynamic collapse [5,9,10]. Symptomatic patients, on average, have worse outcomes than asymptomatic patients [11]. Further evaluation includes determining the presence of an LVOT pressure gradient, often via cardiac catheterization, but is not required for initial diagnostic confirmation. An LVOT gradient at rest of ≥30 mmHg is suggestive of possible LVOT obstruction [1]. Another finding that portends worse outcomes is the presence of SAM of the mitral valve. The anterior movement can impede systolic blood flow out of the left ventricle and can often be the cause of the LVOT obstruction, leading to sudden hypotensive episodes, which can be catastrophic in HOCM patients [1]. 

## Case presentation

The patient was a 77-year-old female with a past medical history of symptomatic HOCM with known SAM, severe LVOT obstruction at rest on recent transthoracic echocardiogram, headaches, and syncopal episodes. Peak LVOT gradient of 128 mmHg and left ventricular wall hypokinesis were also noted. Other past medical history includes hypertension and hyperlipidemia. Her outpatient medications included atorvastatin, metoprolol, and aspirin. The patient does not have a history of overt or witnessed head trauma, but has multiple episodes of syncopal events noted in her medical records with interval CT imaging negative for intracranial hemorrhage (ICH) prior to this presentation. 

**Figure 1:**
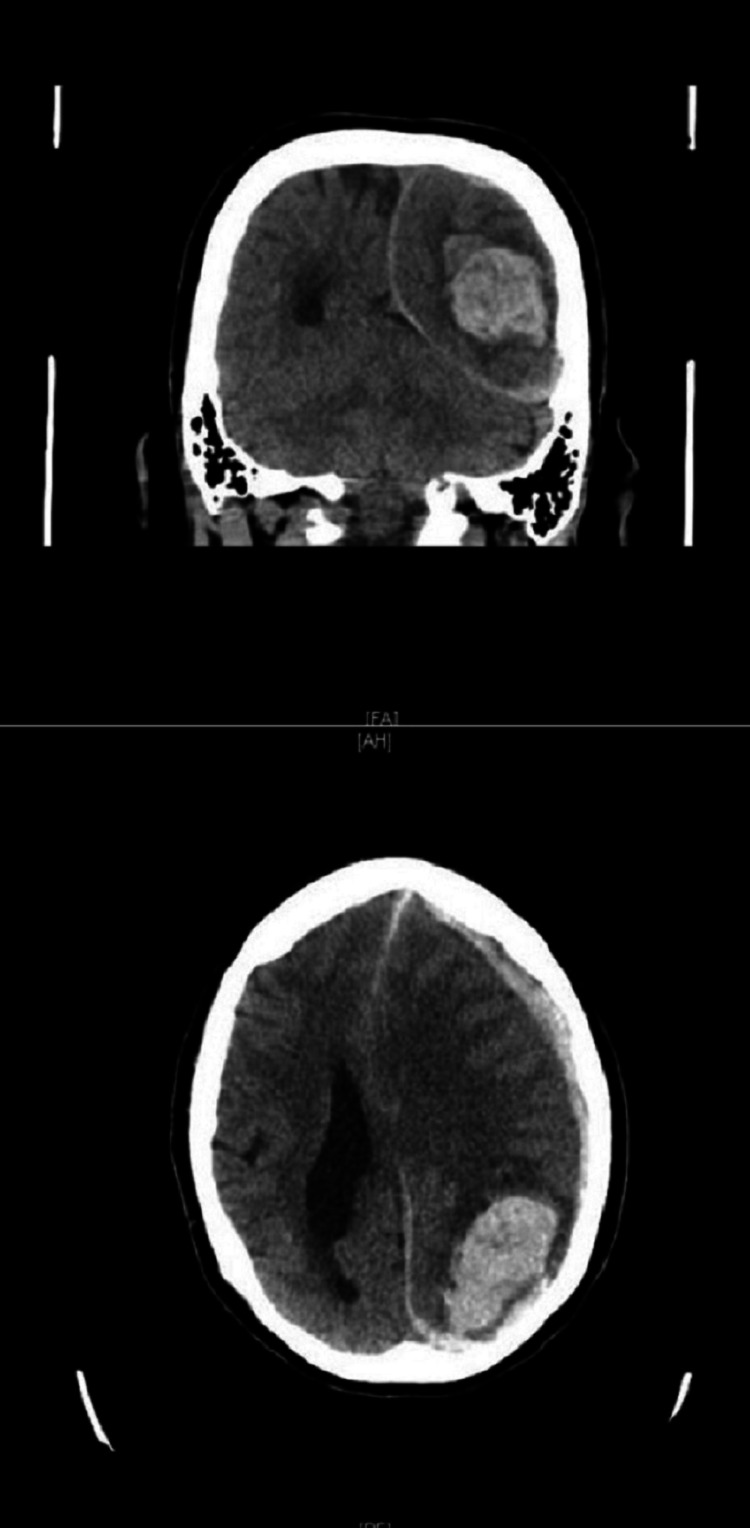
CT Scan of the Head CT scan of the head obtained on presentation in the emergency department, notable for left posterior intracranial hemorrhage. CT, computed tomography.

**Figure 2:**
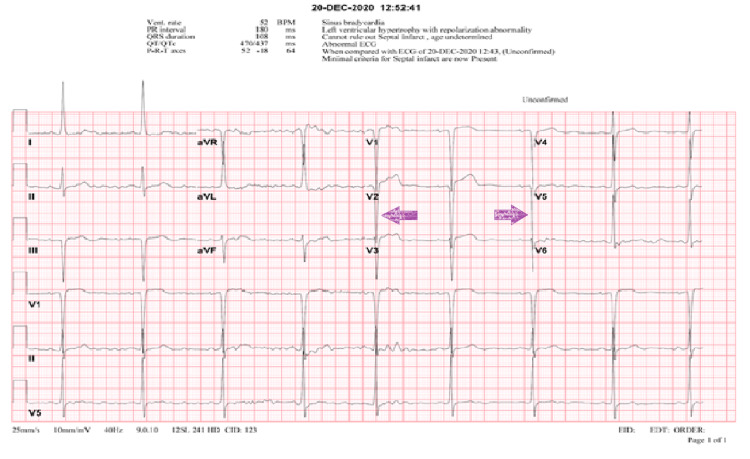
Preoperative EKG Preoperative EKG obtained in the emergency department on presentation. Purple arrows, indicating S wave in V2 and R wave in V5 >35 mm, suggestive of left ventricular hypertrophy. EKG, electrocardiogram.

On 12/20/2020, she presented to the emergency department (ED) with complaints of altered mental status, severe headaches, and generalized weakness. She was found to be in hypertensive emergency with a noninvasive blood pressure of 198/80 mmHg. The patient was unable to follow commands but was responsive to tactile stimulation. Initial head CT (Figure 1) was notable for an acute left parieto-occipital lobar ICH and acute on chronic left subdural hematoma (SDH), with significant mass effect and subfalcine, uncal, and transtentorial herniation (see imaging below). Of note, the CT scan was not able to exclude an existing aneurysm. An electrocardiogram (EKG) obtained on initial assessment indicated sinus bradycardia, anterolateral lead findings suggestive of left ventricular hypertrophy, and 1 mm ST segment elevations in V1, V2, and V3, findings suggestive of herniation (Figure 2). The patient was immediately initiated on antihypertensive therapy with an intravenous (IV) nicardipine infusion in addition to anti-seizure prophylaxis with levetiracetam. Given her active aspirin use, the patient was transfused two units of platelets for reconstitution of platelet function and emergently transported to the operating room (OR) for decompressive craniectomy and hematoma evacuation.

Preoperative airway exam was unremarkable with a Mallampati II airway with normal dentition, thyromental distance, and mouth opening. A preoperative radial arterial line was placed for continuous hemodynamic monitoring. In the OR prior to induction, the patient was administered a 1-L PlasmaLyte fluid bolus and initiated on a remifentanil (0.05 mcg/kg/min) and phenylephrine infusion (25 mcg/min). Following fluid administration, the patient was induced in a controlled fashion with a combination of 2% lidocaine, etomidate, low-dose propofol, a 20 mg esmolol bolus, and remifentanil. Video laryngoscopy was used to reduce sympathetic stimulation and tachycardia. A Cormack-Lehane grade I view was obtained with uncomplicated endotracheal tube placement. Shortly thereafter, the patient was administered rocuronium for paralysis and initiated on volatile anesthesia with 2% sevoflurane. A scalp block was performed using 0.5% plain ropivacaine with 3 mL infiltrated bilaterally over the supraorbital, supratrochlear, and nuchal ridges to similarly prevent a tachycardic response to the placement of the patient’s head in a Mayfield skull clamp. The patient initially responded to the clamp pinning, with a tachycardic and hypertensive response with a heart rate of 112 beats per minute and a peak systolic pressure of 205 mmHg, but quickly resolved to a systolic range of 120-160 mmHg and normal sinus rhythm with a range of 55-70 beats per minute for the remainder of the case. Liberal isotonic fluid administration was utilized to maintain adequate preload. Given the possibility of an aneurysm, the hemodynamic goals were in conflict with the intraoperative management of a HOCM patient, namely the reduction of afterload. Given the severity of the LVOT gradient, the decision was made to maintain appropriate afterload with a systolic goal between 120 mmHg and 140 mmHg. The total duration of the case was 260 min, and the patient did not experience any hypotensive episodes or supposed exacerbation of her HOCM physiology.

## Discussion

Preoperative considerations

Patients who are known to have HOCM/HCM should begin with a preanesthetic consultation, prior to the date of surgery if possible. A preoperative EKG should be obtained to note for baseline electrical changes. The majority of patients will often demonstrate findings suggestive of left ventricular hypertrophy, left atrial enlargement, ST-segment depressions, T-wave inversions, or a left anterior biphasic block [12]. An echocardiogram is highly recommended to assess for the LVOT gradient severity, detect for the presence of SAM, and the degree of LVH present. If echocardiography provides inconclusive results or is technically difficult, other modalities such as cardiac catheterization or a cardiac magnetic resonance imaging (MRI) may be indicated. 

Regarding medication management, chronic medications such as beta blockers and non-dihydropyridine calcium-channel blockers should be continued as they counteract LVOT obstruction while increasing the diastolic perfusion time to the hypertrophied left ventricle. [2,13]. Patients may be on anticoagulation due to atrial fibrillation resulting from left atrial enlargement. Continuation of anticoagulation is dependent on a tailored case-by-case basis that assesses the risk of intraoperative bleeding with the incidence of potential stroke. Preferentially, a bridge from a long-acting or an oral anticoagulant to a shorter-acting medication such as heparin is preferable in the immediate perioperative period [14]. 

Finally, patients with HOCM/HCM are at high risk for adverse cardiac events stemming from arrhythmias such as ventricular fibrillation or sustained ventricular tachycardia [15]. Consequently, implantable cardioverter-defibrillator (ICD) implants are commonly encountered in this patient population when presenting for surgery or medical evaluation. The ICD should be interrogated for proper function, history of firing episodes, battery life, and response of antitachycardia function in response to a magnet. It is also important to discuss with the surgical team the use of electrocautery as electromagnetic interference can affect its function. If electrocautery use is a possibility, it is highly recommended to reprogram the ICD's native function to asynchronous mode. 

Intraoperative considerations

For patients with HOCM undergoing surgical procedures, hemodynamic goals are as such: preload should be maintained to high-normal, rate should be controlled, rhythm should be sinus, contractility should be contained, and afterload should be maintained. Vasodilators, such as dihydropyridine calcium-channel blockers, hydralazine, and propofol, should be avoided intraoperatively. Diuretics and hypotonic fluids should also be avoided, and urine output should be matched with IV fluids, all with the goal of maintaining preload. Negative inotropes, such as beta blockers, are of great benefit in reducing myocardial demand while maintaining diastolic perfusion time. If hypotension is encountered, vasopressors that increase afterload without increasing contractility must be used. Phenylephrine is the primary choice as it increases systemic vascular resistance and causes reflex bradycardia, both effects that help maintain LVOT patency in HOCM/HCM patients. A heart rate of 60-80 beats per minute should be maintained. Low-dose esmolol can be titrated to effect to maintain this heart rate. 

Intraoperative monitoring with invasive arterial access is highly recommended in HOCM patients, especially with additional risk factors present including but not limited to reduced ejection fraction, pulmonary hypertension, and history of clinically significant arrhythmias such as rapid atrial fibrillation, ventricular tachycardia, or ventricular fibrillation. Placement of arterial access catheter is recommended before induction. Transesophageal echocardiography may be indicated in patients who are likely to have large shifts in their hemodynamic parameters. It can also be used for monitoring of SAM or evaluation of a patient experiencing hemodynamic instability. Central venous catheters may be indicated if large-bore peripheral access cannot be obtained. However, routine placement for central venous monitoring is not recommended. 

Postoperative monitoring and recovery

**Figure 3:**
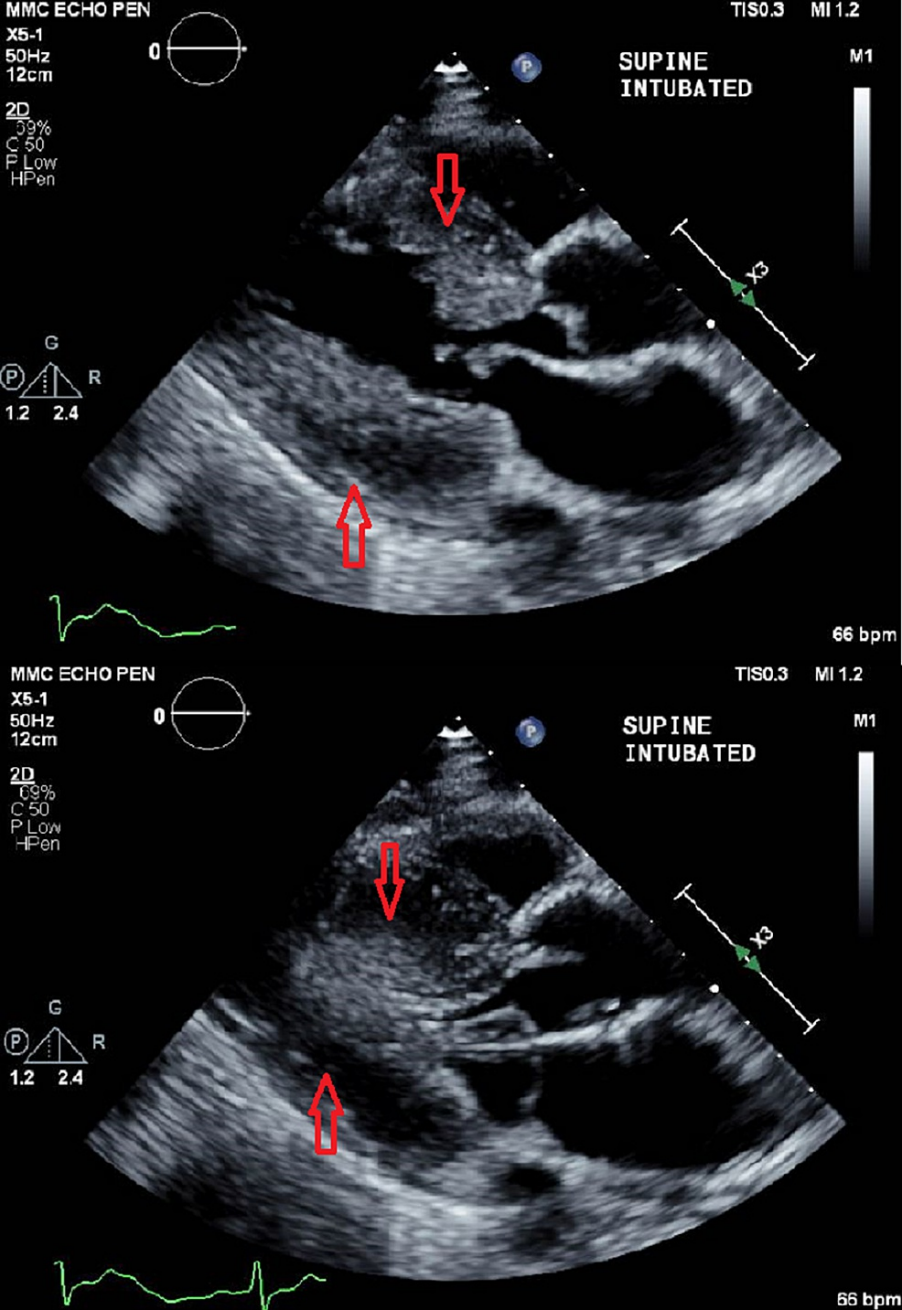
Transthoracic Echocardiogram Still images of a transthoracic echocardiogram obtained postoperatively to highlight the severity and extent of left ventricular hypertrophy and left ventricular cavity size during diastole and systole. Red arrows point to thickened anterior and inferior left ventricular walls.

**Figure 4:**
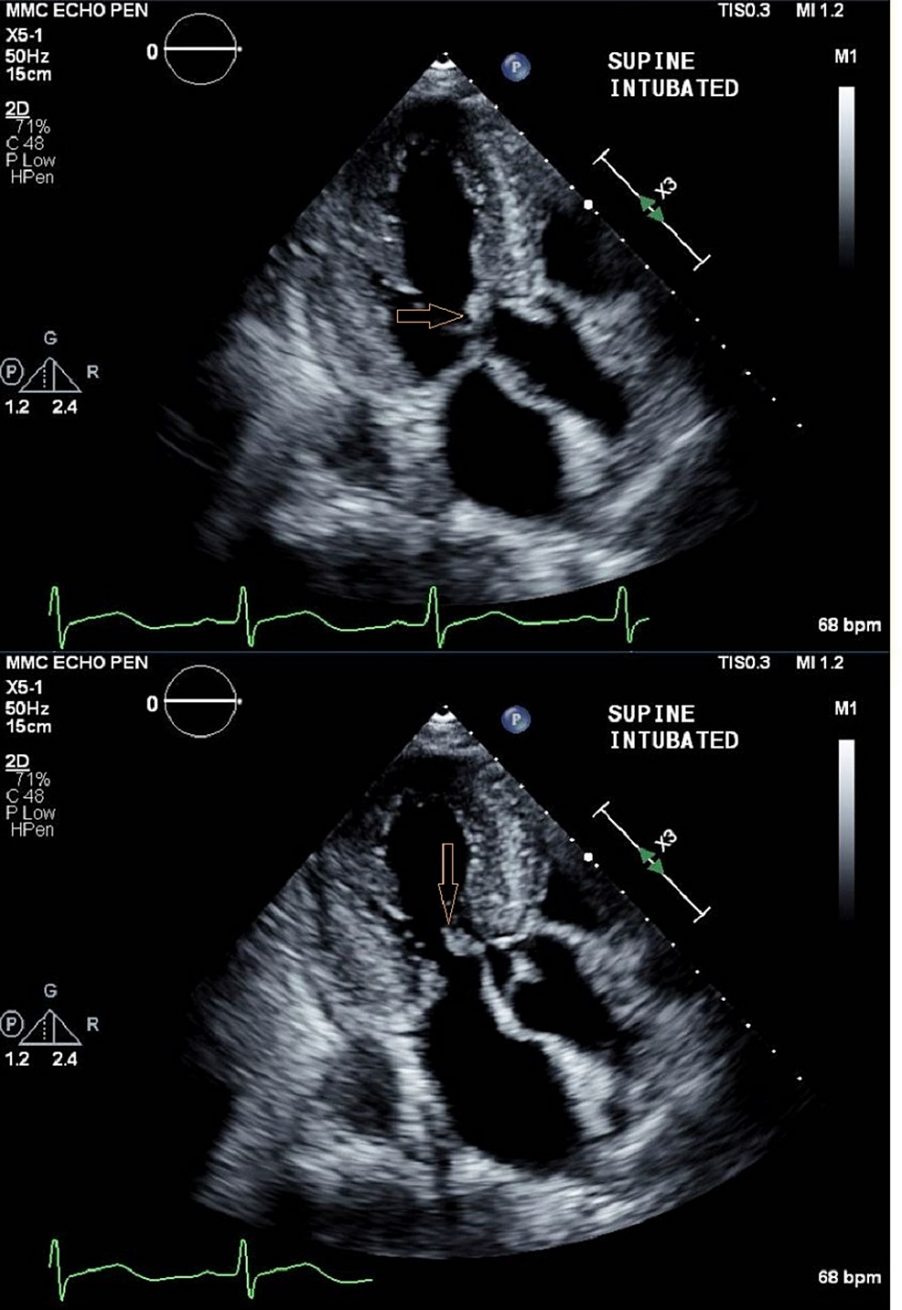
Transthoracic Echocardiogram Transthoracic echocardiogram obtained postoperatively to highlight the extent of left ventricular outflow tract obstruction. In the bottom image, the anterior mitral valve leaflet is highlighted during diastole. In the top image, the anterior mitral valve leaflet is displaced into the left ventricular outflow tract, resulting in restriction of forward blood flow.

Postoperatively, the patient was transferred intubated to the neurosurgical intensive care unit (NSICU), with a phenylephrine infusion to maintain appropriate afterload. Suggamadex was administered in the NSICU to assess the patient’s spontaneous ventilatory function and interval neurological examinations. A postoperative echocardiogram was performed, notable for severely hypertrophied left ventricle (Figure 3) as well as the presence of SAM of the mitral valve (Figure 4). Her ICU course was complicated by platelet dysfunction, seizures, serratia pneumonia, and poor renal function. The phenylephrine infusion was eventually weaned a few hours after arrival to the ICU. The patient was unable to be weaned from the ventilator, resulting in a tracheostomy and gastrostomy tube placement. Neurological examination was notable for persistent right-sided hemiplegia. During the admission a postoperative cerebral angiogram was performed to determine the cause of the patient's ICH. At this time, the presence of an aneurysm or other vascular malformations were definitively excluded. Embolization of the middle meningeal artery was performed to reduce rebleeding risk and recurrence of the patient's subdural hemorrhage. The patient was discharged to a subacute rehabilitation facility on 1/9/2021, postoperative day 20, in stable condition.

## Conclusions

In this case report, we discuss the emergent management of a patient with severe HOCM presenting with an acute ICH complicated by an acute on chronic SDH for decompressive craniectomy, given the conflicting hemodynamic management goals for a HOCM patient vs a patient with an ongoing intracranial bleed and suspected cerebral aneurysm. We prioritized maintenance of afterload in this patient given the potentially lethal risk of cardiovascular collapse if LVOT obstruction was encountered.
